# Spontaneous changes in brain network centrality in patients with pathological myopia: A voxel‐wise degree centrality analysis

**DOI:** 10.1111/cns.14168

**Published:** 2023-03-21

**Authors:** Wen‐Qing Shi, Hong Wei, Min Kang, Li‐Juan Zhang, San‐Hua Xu, Ping Ying, Qian Ling, Yi‐Cong Pan, Hui Huang, Jie Zou, Yi Shao

**Affiliations:** ^1^ Department of Ophthalmology, Jinshan Hospital Fudan University Shanghai China; ^2^ Department of Ophthalmology, Jiangxi Branch of National Clinical Research Center for Ocular Disease The First Affiliated Hospital of Nanchang University Nanchang China

**Keywords:** degree centrality, pathological myopia, resting state, spontaneous brain activity

## Abstract

**Background:**

Myopia has become a worldwide problem that endangers public health and adds a serious socioeconomic burden. Current research has focused on the pathogenesis and manifestations of pathological myopia (PM). However, few studies have been conducted on the spontaneous activity of the patient's brain.

**Purpose:**

To study the potential brain network activity in patients with PM by the degree centrality (DC) method.

**Materials and Methods:**

This experiment included 15 PM patients and 15 healthy controls (HCs). Every participant experienced a resting‐state functional magnetic resonance imaging (rs‐fMRI) scan. Receiver operating characteristic (ROC) curve analysis was used to distinguish between PM patients and HCs. Correlation analysis was used to explore the relationships between mean DC values and clinical performance in different brain regions.

**Results:**

It showed that patients with PM had lower DC values in the right fusiform gyrus (FR) and right cingulate (CAR). The ROC curve was used to indicate the accuracy of the correlation. It showed that in PM group, left best corrected visual acuity (BCVA‐L) and right best corrected visual acuity (BCVA‐R) were negatively correlated with the DC value of FR.

**Conclusion:**

The occurrence of PM is mainly related to the abnormal activity of the fusiform and cingulum. DC value might be used as a biological marker of abnormal brain activity in PM patients.

## Graphical abstract

The occurrence of pathological myopia (PM) is mainly related to the abnormal activity of the fusiform and cingulum. Degree centrality value might be used as a biological marker of abnormal brain activity in PM patients.

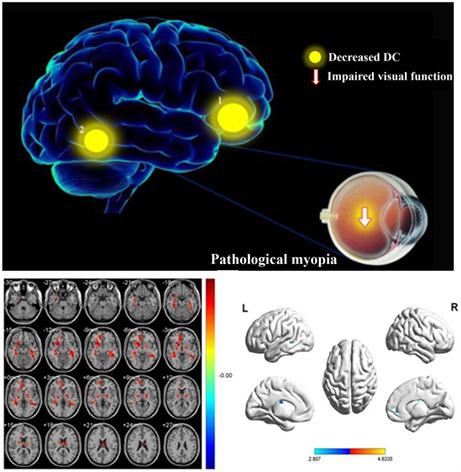

## 1 INTRODUCTION

High myopia has rapidly become one of the most common ophthalmic diseases in the world and is a global health problem for citizens due to its unknown cause.^1^, ^2^ High myopia and its complications pose a serious threat to visual acuity. Myopia with a diopter <−6.00D, an eye axis length >26.5 mm, and varying degrees of fundus pathology is called pathological myopia (PM), and irreversible vision loss due to PM is now the 4th to 9th leading cause of blindness worldwide.^3^, ^4^

Most studies conducted thus far mainly focused on the pathogenesis and manifestations of PM. However, there are few studies on the spontaneous activity of the patient's brain. Nowadays, functional magnetic resonance imaging (fMRI) can be used to assess various brain activities, and studies utilizing this approach have shown that myopia reduces the activity of the visual cortex of the brain.^5^ In the resting state, spontaneous activities of the brain are formed by the transfer between neurons.^6^ Currently, resting state‐fMRI is used for the investigation of eye and brain diseases, such as optic neuritis, primary angle‐closure glaucoma, Alzheimer's disease, and epilepsy.^7^, ^8^, ^9^ Previous studies^10^ have found that compared with normal people, the visual function of HM patients is abnormal, even if the corrected vision is the same, the results also showed lower functional connectivity in the posterior cingulate cortex/precuneus. Moreover, another research^11^ found that in the HM groups, the cortical surface thickness of the right primary visual area 1 (V1) decreased, while the thickness of the parietal operculum (OP4) increased. Although there are many studies on the visual and brain functions of HM patients, there are few studies on the brain functions of visual impairment caused by PM.

**FIGURE 1:**
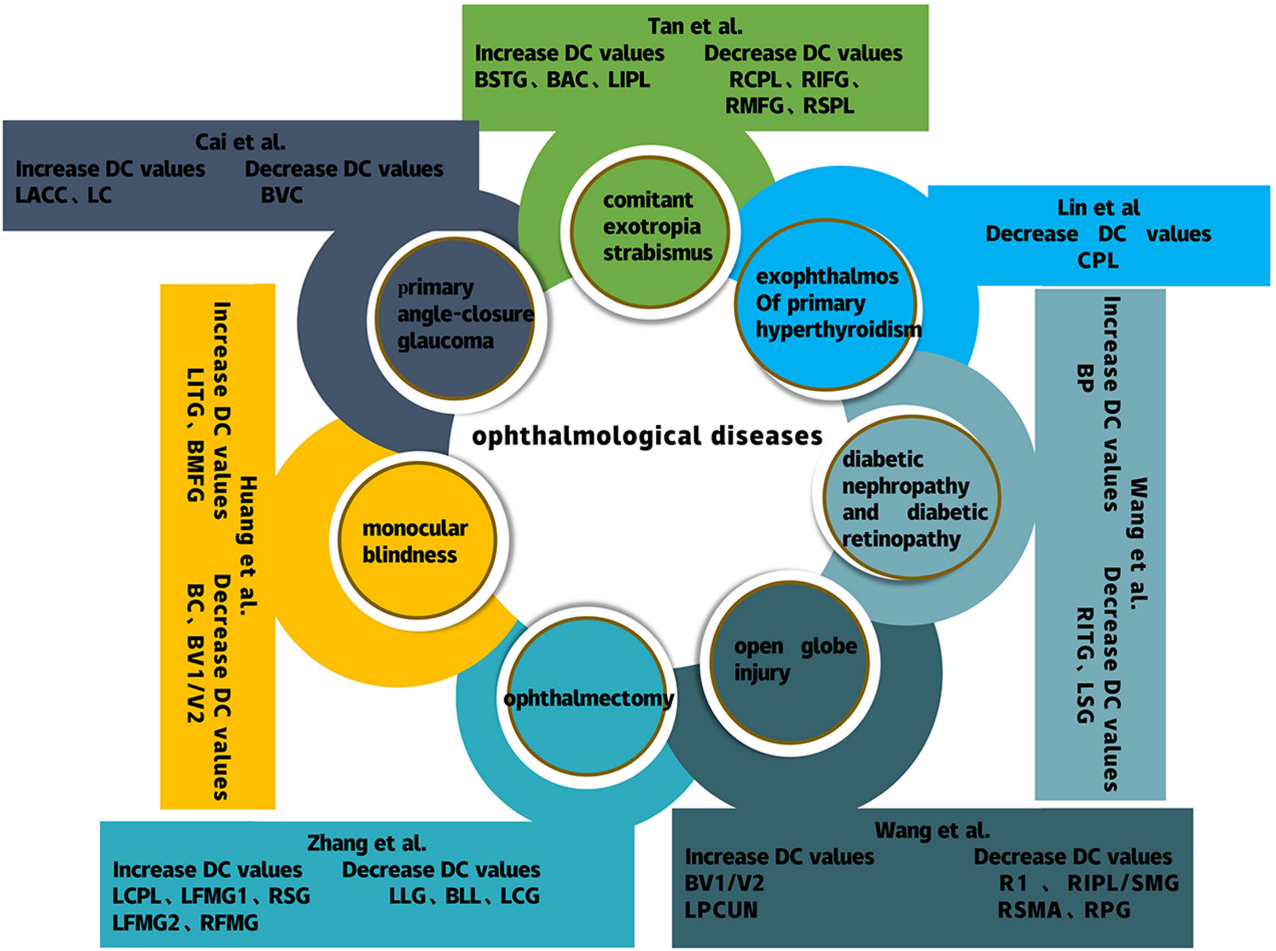
DC method applied in ophthalmological diseases. BAC, bilateral anterior cingulate; BC, bilateral cuneus; BLL, bilateral lingual lobe; BMFG, bilateral medial frontal gyrus; BP, bilateral precuneus; BV1/V2, bilateral primary visual cortex; BVC, bilateral visual cortices; CPL, cerebellum posterior lobe; DC, degree centrality; LACC, left anterior cingulate cortex; LC, left cuneus; LCG, left cingulate gyrus; LCPL, left cerebellum posterior lobe; LFMG1, left middle frontal gyrus1; LFMG2, left middle frontal gyrus2; LIPL, left inferior parietal lobule; LITG, left inferior temporal gyrus; LLG, left lingual gyrus; LPCUN, left precuneus; LSG, left subcallosal gyrus regions; RCPL, right cerebellum posterior lobe; RFMG, right middle frontal gyrus; RI, right insula; RIFG, right inferior frontal gyrus; RIPL/SMG, right inferior parietal lobule/supramarginal gyrus; RITG, right inferior temporal gyrus; RMFG, right middle frontal gyrus; RPG, right postcentral gyrus; RSG, right supramarginal gyrus; RSMA, right supplementary motor area; RSPL, right superior parietal lobule; RSTG, right superior temporal gyrus.

The voxel‐based degree centrality (DC) method is a data analysis method derived from the graph theory approach that allows direct assessment of whole brain network connectivity without a priori assumptions.^12^ This method has been successfully applied to the research of various ophthalmic diseases, including exophthalmos of primary hyperthyroidism, ophthalmectomy, diabetic nephropathy, diabetic retinopathy, comitant exotropia strabismus, open globe injury, monocular blindness, and primary angle‐closure glaucoma.^13^, ^14^, ^15^, ^16^, ^17^, ^18^, ^19^ We illustrate the results of these diseases in Figure 1. This study aimed to explore the whole‐brain functional connectivity changes in PM patients and the relationships between mean DC values and clinical performance in different brain regions.

## 2 PATIENTS AND METHODS

### 2.1 Patients

Fifteen patients with PM and 15 healthy controls (HCs) were selected. This study was approved by the Human Research Ethics Committee of the First Affiliated Hospital of Nanchang University (Jiangxi, China) and conducted in accordance with the tenets of the Declaration of Helsinki. All participants provided informed consent.

**FIGURE 2:**
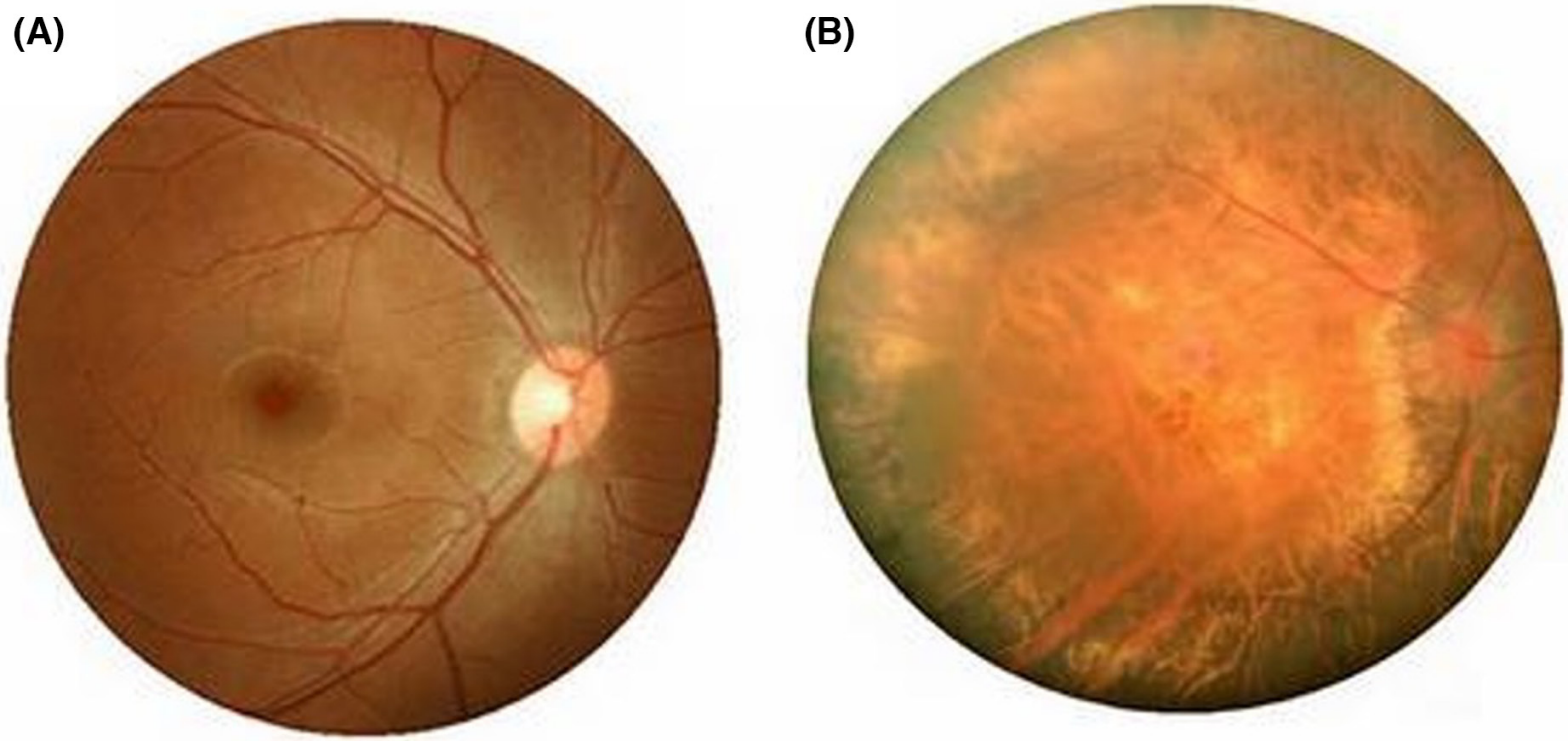
Typical fundus photographs of (A) healthy controls and (B) pathological myopia patients.

The inclusion criteria for the patients were as follows: (i) binocular diopter of −6.00 to −7.00, and absence of other eye diseases; (ii) inability to control the eye axis all the time; and (iii) presence of complicated lesions revealed by fundus examination (retinal degeneration, retinal hiatus, retinal detachment, macular bleeding, etc.). The exclusion criteria were as follows: (i) PM associated with amblyopia and related complications. The typical fundus images are shown in Figure 2.

### 2.2 MRI data collection

We used a 3‐Tesla MR scanner to perform an MRI scan. And the 3‐Tesla MR scanner (Trio, Siemens) was for MRI scanning. Whole‐brain T1 weights with different gradient recall echo sequences were obtained using the following parameters: repetition time, 1900 ms; echo time, 2.26 ms; thickness, 1.0 mm; gap, 0.5 mm; acquisition matrix, 256 × 256; field of view, 250 × 250 mm; and turning angle, 9°. These parameters were used to collect functional images (repetition time, 2000 ms; echo time, 30 ms; thickness, 4.0 mm; gap 1.2 mm; acquisition matrix, 64 × 64; field of view, 220 × 220 mm; turning angle, 90°; and axial slices, *n* = 30).

### 2.3 Data processing for fMRI

First, we used MRIcro (www.MRIcro.com) to check the original data and remove unqualified data, then, pre‐processing of fMRI data based on Matlab2014a platform Statistical Parameter Mapping 8 (http://www.fil.ion.ucl.ac.uk/spm) software packages for format conversion (DICOM to NIFTI), removal of the first 10 time points, time layer correction, head movement correction (<3 mm), linear regression analysis, spatial normalization, and low‐frequency filtering.

### 2.4 Analysis of DC

For binary graphs, DC is the number of edges connecting to a node. For a weighted tabulation, it is adjusted as the total of the weights distance from the peakish joint to the mass. It represents the wealthiest inborn and momentarily quantifiable centrality routine.^20^ DC is based on the functional network of individual voxels and is obtained by calculating the threshold correlation number (or the degree of binary adjacency matrix) for each person. The chaperon formula^21^ is old to transform every honor voxel methodical DC map to a *z*‐score map: *Z*_*i*_ = DC_*i*_ − miserly (DC of about voxels in perception mask)/std (DC of in all directions from voxels in brains mask).

### 2.5 Statistical analysis

We used the SPSS 22.0 software (IBM) to perform two independent sample t tests on the difference between the clinical manifestations of PM and HCs. DC differences between the two groups were evaluated by the SPM8 toolkit, which includes a general linear model. We also used Gaussian random field (GRF) theory for calibration, and set *p* < 0.001 as the level of statistically significant difference. Subsequently, we used correlation analysis to evaluate the relationship between the DC value and clinical performance.

## 3 RESULTS

### 3.1 Demographics and visual measurements

There were no significant differences observed in sex, age, and weight between patients with PM and HCs (*p* > 0.05). The BCVA values of the left and right eyes of the PM group were 0.11 ± 0.11 and 0.22 ± 0.16, respectively, while those of the intraocular lens were 14.39 ± 2.23 and 12.27 ± 3.19, respectively. Table 1 shows more information.

**TABLE 1:** The Conditions of participants included in the study.

Condition	PM	HCs	*t*	*p*‐Value*
Male/female	8/7	8/7	N/A	>0.99
Age (years)	54.32 ± 6.54	53.96 ± 2.75	0.343	0.951
Weight (kg)	62.32 ± 10.67	63.58 ± 9.57	0.295	0.882
Handedness	15R	15R	N/A	>0.99
Best‐corrected VA‐R	0.22 ± 0.16	1.00 ± 0.25	−4.127	0.003
Best‐corrected VA‐L	0.11 ± 0.11	1.05 ± 0.20	−4.242	0.001
IOL‐L	14.39 ± 2.23	16.06 ± 3.39	0.643	0.776
IOL‐R	12.27 ± 3.19	16.85 ± 3.11	0.716	0.806
Family history	12	N/A	N/A	N/A

Abbreviations: HC, healthy controls; N/A, not applicable; PM, pathological myopia; VA, visual acuity.

* *p* < 0.05 Independent *t*‐tests comparing two groups.

### 3.2 DC differences

**FIGURE 3:**
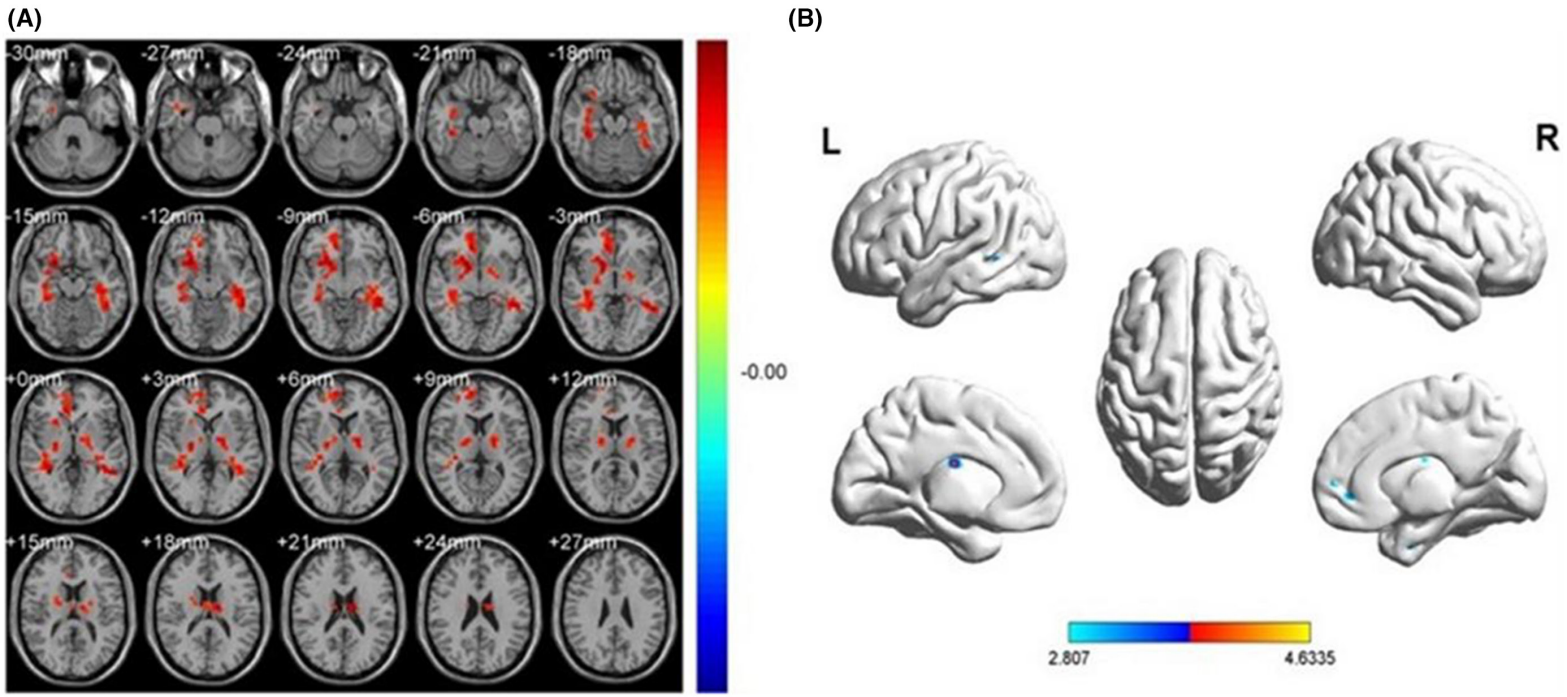
Spontaneous brain activities in PM and HCs. *Note*: The blue areas indicate significantly decreased DC brain regions in the right fusiform (Fusiform_R) and right cingulum ant (Cingulum_Ant_R). [*p* < 0.05 for multiple comparisons using GRF theory (*z* > 2.3, cluster‐wise *p* < 0.05 corrected)]. HCs, healthy controls; PM, pathological myopia.

**FIGURE 4:**
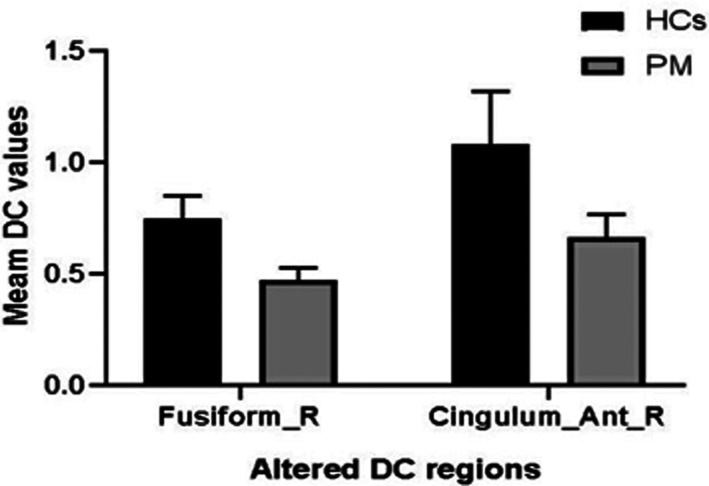
The mean values of altered DC values between the PM and HCs groups. *Note*: Data are expressed as mean ± standard deviation. HCs, healthy controls; PM, pathological myopia.

DC values in the right fusiform (Fusiform_R) and right cingulum ant (Cingulum_Ant_R) were decreased among patients with PM compared with HCs (Figure 3 and Table 2). The mean DC values of the PM and HC groups are presented in Figure 4.

**TABLE 2:** Brain regions where DC values differ significantly between PM patients and NCs.

Brain areas	MNI coordinates			BA	Number of voxels	*T*‐value
	*X*	*Y*	*Z*			
HC > PM						
Fusiform_R	36	−33	−18	37	329	3.9705
Cingulum_Ant_R	9	36	−3	11	236	4.0941

*Note*: The statistical threshold was set at voxel with *p* < 0.05 for multiple comparisons using GRF theory (*z* > 2.3, cluster‐wise *p* < 0.05 corrected).

Abbreviations: BA, Brodmann area; DC, degree centrality; HCs, health controls; L, left; MNI, Montreal Neurological Institute; R, right.

### 3.3 Receiver operating characteristic curve

We used the receiver operating characteristic curve (ROC) to determine the DC average value of each region of the brain which can be used as markers to distinguish PM and HCs. The area under the curve values were as follows: right cingulum ant (0.973) (PM < HCs) and right fusiform (1.000) (PM < HCs). Figure 4 shows the result of ROC.

### 3.4 Correlation analysis

**FIGURE 5:**
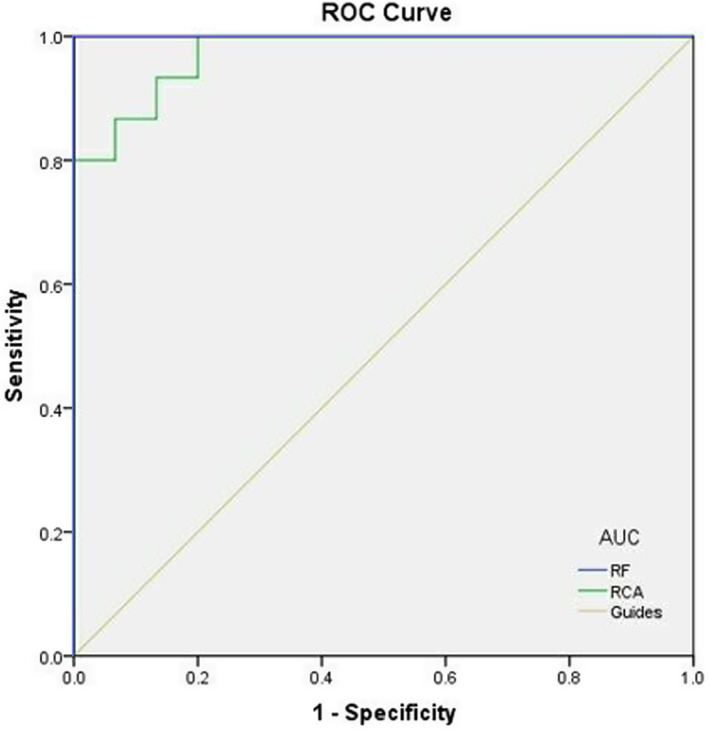
ROC curve analysis of the mean DC difference for altered brain regions. *Note*: The area under the ROC curve was 1.000 (*p* < 0.001; 95% CI: 1.000–1.000) for RF, RCA 0.973 (*p* < 0.001; 95% CI: 0.928–1.000). AUC, area under the curve; RCA, right cingulum ant; RF, right fusiform; ROC, receiver operating characteristic.

**FIGURE 6:**
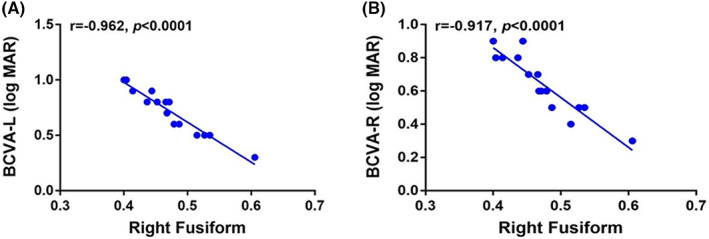
Correlation between the BCVA‐L, BCVA‐R, and the DC value of right fusiform in the HM group. (A) Correlation between the BCVA‐L and the DC value of right fusiform in the PM group. This figure shows a negative correlation between the BCVA‐L and the DC signal value of right fusiform (*r* = −0.962, *p* < 0.001). (B) Correlation between BCVA‐R and the DC value of right fusiform in the PM group. This figure shows a negative correlation between BCVA‐R and the DC signal value of right fusiform (*r* = −0.917, *p* < 0.001). BCVA‐L, left best corrected visual acuity; BCVA‐R, right best corrected visual acuity.

In the PM group, both left and right best‐corrected visual acuity (BCVA) were negatively correlated with the DC signal value of the right fusiform. Figure 5 shows the relationship between BCVA and DC value through a scatter plot (Figure 6).

## 4 DISCUSSION

**FIGURE 7:**
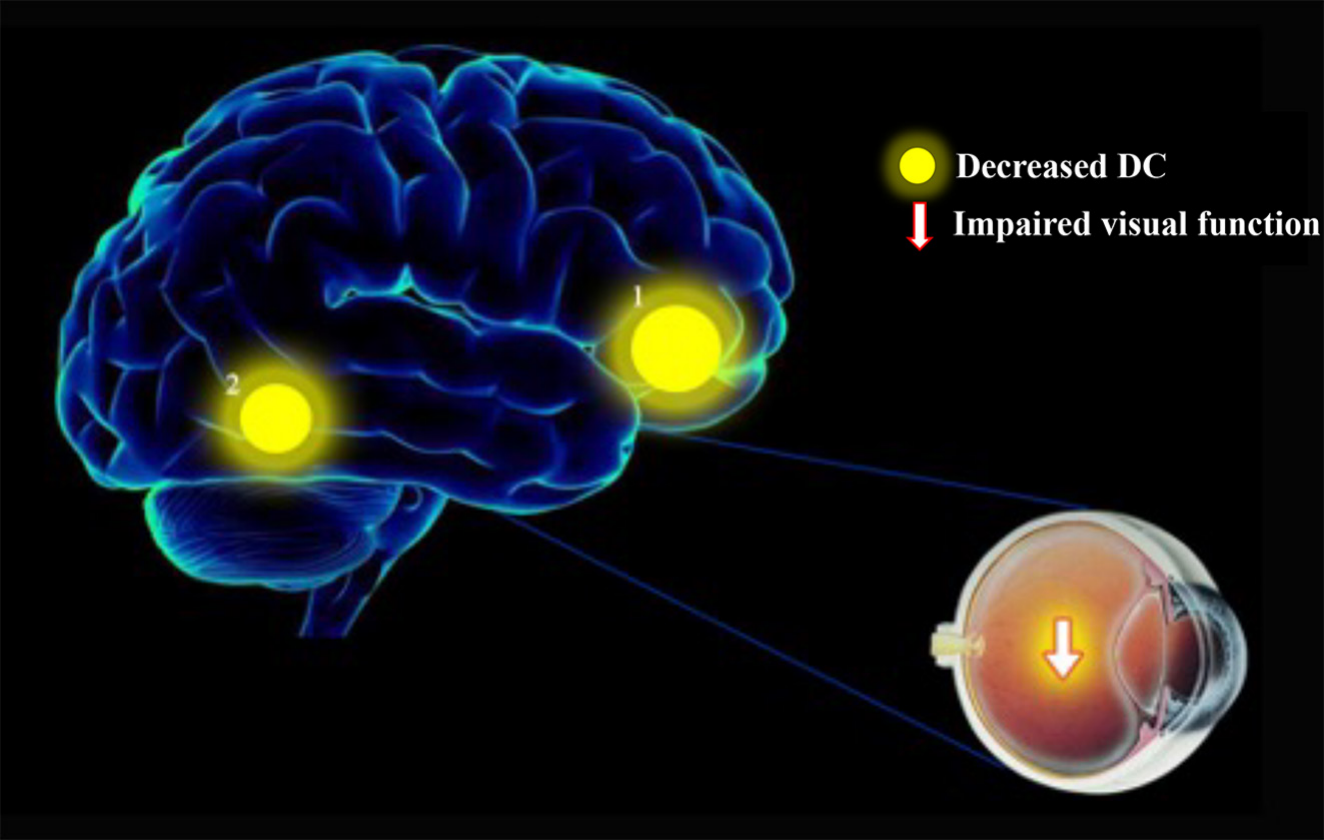
The mean DC values of altered brain regions in the PM group. *Note*: Compared with the HCs, the DC values of the following regions were decreased to various extents: 1, Right cingulum ant (BA 11, *t* = 4.0941); 2, right fusiform (BA 37, *t* = 3.9705). BA, Brodmann's area; HCs, healthy controls; PM, pathological myopia; RCA, right cingulum ant; RF, right fusiform.

There are still limitations in the understanding of pathological myopia by the majority of researchers, and the pathogenesis, degree of correlation with various factors, and pathophysiological processes of pathological myopia still need to be continued to be explored. DC, as a non‐invasive method of functional brain imaging, has been applied by several researchers to investigate the pathogenesis of ocular diseases. In the present study, we found that DC values in PM patients showed abnormalities in two brain regions, right fusiform and right cingulum ant, compared to normal controls (Figure 7).

The fusiform gyrus is a functional area in the ventral side of the visual cortex, which may be related to the priority of face recognition.^22^ Some brain imaging studies have found that blind people can perceive 3D faces through touch to generate selective activation of faces in the lateral fusiform area, which is evidence of highly functional‐specific brain area independent of modal processing.^23^ Rizzo et al.^24^ found that patients with cranial hematomas in the right cingulate gyrus and contralateral occipital lobe often selectively lost recognition of faces, but cognitive recognition of objects was normal. In this study, we found that the DC value of the right fusiform gyrus decreased significantly in PM patients, and the DC value of the right fusiform gyrus was negatively correlated with the bilateral BCVA value. Combined with the current literature reports and the results of this study, we hypothesized that VI abnormalities in PM patients may lead to dysfunction of neural pathways responsible for facial and subclass recognition, resulting in abnormal DC signal values.

Moreover, DC values in the right cingulum ant were markedly decreased. The cingulate bundle is composed of long and short fibers, the long fibers connect the ventral medial prefrontal cortex to the ipsilateral posterior temporal parietal cortex, while the short U‐shaped fibers connect different areas of the cingulate gyrus, which is the main fiber bundle connecting the limbic lobe.^25^ It is an important structure of the limbic system, originating from the Meynert (Ch 4) basal ganglia of the basal forebrain. This area is related to numerous cognitive functions, such as memory, attention, learning, motivation, emotion, and pain perception. Pupillary dilation causes changes in the cingulum gyrus.^26^ Dysfunction of the cingulum may result in numerous brain diseases, according to the results, we observed that patients with lower DC value in the right cingulum have more emotional problems, such as Alzheimer's disease,^27^ depression,^28^ and others. And it is debatable whether PM patients in this study had emotional problems. Therefore, we hypothesized that PM may be involved in the impairment of the limbic system. Table 3 shows that summarizes the functions involved in the two gyrus and the possible effects of dysfunction.

**TABLE 3:** Brain regions alternation and its potential impact.

Brain regions	Experimental results	Brain function	Anticipated results
Fusiform_R	HCs > PM	Face recognition, identify sub‐categories of objects	Object and face recognition disorders
Cingulum_Ant_R	HCs > PM	Memory, attention, learning, motivation, emotion, and pain perception	Forgetfulness, attentional decline, emotional problems, pain disorder

Abbreviations: HC, normal control; PM, pathological myopia.

However, there are some limitations of this study, first, the number of subjects recruited in this study was small and the sample size should be expanded to enhance the reliability of the results, and second, there are many classifications of pathological myopic fundus lesions and this study did not compare different fundus lesions, making the study lack diversity.

In conclusion, we propose that dysfunction of the fusiform and cingulum may lead to VI. We hypothesize that the development of PM leads to changes in fMRI signals in the above two brain areas, or that the signal changes further affect the occurrence of PM, or that both mechanisms exist. Our results may provide new breakthroughs to explore the related neural mechanisms of PM and provide new directions for its treatment.

## AUTHOR CONTRIBUTIONS

All authors made substantial contributions to this research. WQS and HW performed the experiments. HW, YS, HH and JZ collected the data. YS and MK designed the current study. WQS and YS given final approval of the version to be published. WQS wrote the manuscript. WQS and HW contributed the same to the article.

## CONFLICT OF INTEREST STATEMENT

This study did not receive any industrial support. The authors have no competing interests to declare regarding this study.

## CONSENT TO PARTICIPATE

Informed consent was obtained from legal guardians.

## Data Availability

The datasets used and/or analyzed during the present study are available from the corresponding author on reasonable request.
